# A Novel Multi-Objective Electromagnetic Analysis Based on Genetic Algorithm

**DOI:** 10.3390/s19245542

**Published:** 2019-12-15

**Authors:** Shaofei Sun, Hongxin Zhang, Liang Dong, Xiaotong Cui, Weijun Cheng, Muhammad Saad Khan

**Affiliations:** 1 School of Electronic Engineering, Beijing University of Posts and Telecommunications, Beijing 100876, China; sfsun@bupt.edu.cn (S.S.); dongliang@163.com (L.D.); cuixiaotong@bupt.edu.cn (X.C.); 2 Communication and Electronic Engineering Institute, Qiqihar University, Qiqihar 161006, China; 3 School of Information Engineering, Minzu University of China, Beijing 100081, China; weijuncheng@muc.edu.cn; 4 Electrical Engineering Department, Bahauddin Zakariya University, Multan 60000, Pakistan; saadkhan9@gmail.com

**Keywords:** Advanced Encryption Standard (AES), correlation electromagnetic analysis, genetic algorithm, multi-objective optimization

## Abstract

Correlation electromagnetic analysis (CEMA) is a method prevalent in side-channel analysis of cryptographic devices. Its success mostly depends on the quality of electromagnetic signals acquired from the devices. In the past, only one byte of the key was analyzed and other bytes were regarded as noise. Apparently, other bytes’ useful information was wasted, which may increase the difficulty of recovering the key. Multi-objective optimization is a good way to solve the problem of a single byte of the key. In this work, we applied multi-objective optimization to correlation electromagnetic analysis taking all bytes of the key into consideration. Combining the advantages of multi-objective optimization and genetic algorithm, we put forward a novel multi-objective electromagnetic analysis based on a genetic algorithm to take full advantage of information when recovering the key. Experiments with an Advanced Encryption Standard (AES) cryptographic algorithm on a Sakura-G board demonstrate the efficiency of our method in practice. The experimental results show that our method reduces the number of traces required in correlation electromagnetic analysis. It achieved approximately 42.72% improvement for the corresponding case compared with CEMA.

## 1. Introduction

With the development of Internet of Things (IoT) technology, ever more smart home devices are appearing in people’s lives, such as mobile phones, smart door locks, smart televisions, sensor networks and many other things. Their security issues are becoming increasingly prominent. Unintentional physical leakage of these devices can be acquired by sensors. Side-channel analysis [1] exploits these unintentional physical leakage to obtain sensitive information. Up to now, none of the smart home devices are able to prevent the leakage of information through different side channels. Side-channel analysis has attracted a great attention since timing attacks were introduced by Kocher in 1996 [2]. A variety of efficient approaches have proposed in the past 20 years, such as differential power analysis (DPA) [3], correlation power analysis (CPA) [4], template analysis (TA) [5,6], fault analysis (FA) [7], electromagnetic analysis (EMA) [8,9,10,11,12] and so on. For DPA, plenty of power traces are statistically computed to reveal the power consumption difference caused by different intermediate values and then the difference is used to recover the key. For CPA, the Pearson correlation coefficient is used to measure the relationship between the power traces and intermediate values’ hamming weight or hamming distance model. There is a larger correlation coefficient than others when the right key occurs. In template analysis [1], power traces are characterized by a multivariate normal distribution. This usually consists of two steps: the first step is to create a template of a device’s operation using a copy of the protected device and the second step is to apply the template to the attack traces to recover the key. Fault analysis uses the faulty calculations of the cryptographic algorithms to recover the key [13]. EMA is similar to power analysis, but it uses electromagnetic traces to help recover the key. For EMA, correlation electromagnetic analysis (CEMA) is a traditional and prevailing method. Its performance is much better than other ways in traditional electromagnetic side-channel analysis.

In recent years, many artificial intelligence methods have emerged in side channel analysis. On the one hand, to date most artificial intelligence methods are profiling attacks. Machine learning was first applied to side-channel analysis by Hospodar et al. in [14] and they applied a least squares support vector machine (LS-SVM) to classify intermediate values. In [15], the author used machine learning techniques to deal with high-dimensional feature vectors in side-channel analysis. SVM was used to classify arithmetic operations by electromagnetic leakage in [16]. In [17], the author found that convolutional neural networks were more suitable for side-channel analysis scenarios than some other machine-learning techniques without preprocessing. On the other hand, there are some new directions different from the previous. Zhang et al. [18] employed simple genetic algorithm to turn the key searching problem into a correlation coefficient optimization problem based on a non-profiling attack which is called SGA-CPA. In [19], Ding et al. improved Zhang’s method and proposed the multiple sieve method to overcome premature convergence.

In this paper, we proposed a novel multi-objective electromagnetic analysis based on genetic algorithm to recover the key. Most machine-learning methods are profiling attacks so that they need a copy of the protected device to obtain enough traces. Power analysis or fault analysis is an invasive way to obtain sensitive information that will modify the protected device. Electromagnetic analysis is a non-invasive way to obtain sensitive information. In this way, a malicious adversary neither needs to have a copy of the protected device nor to modify the protected device, so it is more practical in real-world situations.

The main contributions of the paper are as follows:

(1) We applied multi-objective optimization to correlation electromagnetic analysis to take full advantage of information. A genetic algorithm is the most popular heuristic approach to multi-objective optimization problems. So our method combines these two ways to recover the key which has seldom been studied in side-channel analysis.
(2) In the past, traditional correlation electromagnetic analysis only focused on one byte of the key which may lose information and efficiency, because other bytes also contains partial information related to the secret key. So all bytes of the key are used in our method to take full advantage of information.
(3) We also modify genetic algorithm to make our method more applicable in different scenarios. We add two operators—sort and sieve—to a genetic algorithm. For sort operator, we add it after selection, crossover and mutation. This operation will sort subkey candidates in descending order so that better candidates in different groups can be combined together with greater probability. For sieve operator, better key candidates selected in every generation will be sieved and recombined to obtain the best key candidate.

We take AES-128 for instance to illustrate our method. First, all 128 bits of the key are initialized as the genetic algorithm’s chromosome code; then, we compute the correlation between every byte and electromagnetic traces; next, we use multi-objective optimization to optimize the correlation between the entire key bits and traces. Finally, we get the max correlation coefficient corresponding to the right key after many generations’ optimization.

The remainder of the paper is organized as follows: in Section 2, we present related works and basic knowledge about the cryptographic algorithm, genetic algorithm and multi-objective optimization used in our experiment. In Section 3, we introduce a novel electromagnetic analysis method and the experimental platform in detail. The performance of different methods on different trace sets is shown in Section 4. Finally, we conclude the paper in Section 5.

## 2. Related Works and Preliminaries

### 2.1. Related Works

Multi-objective optimization has made great achievements in other fields. For example Li et al. [20] proposed an energy-aware multi-objective optimization algorithm (EA-MOA) for solving the hybrid flow shop-scheduling problem with consideration of the setup energy consumptions. Through the analysis of the experimental results, the EA-MOA algorithm was found to be more effective than other efficient algorithms in literature. Maryam et al. [21] proposed a multi-objective Particle Swarm Optimization(PSO)-based method named RFPSOFS (Ranked Feature PSO Feature Selection) that ranks the features based on their frequencies in the archive set. RFPSOFS improve feature selection methods using single objective optimization algorithm and obtained a remarkable performance. Du et al. [22] employed multi-objective ant lion optimization to optimize the initial weights between layers and thresholds of the Elman neutral network in the optimization module to overcome the drawbacks of single-objective optimization algorithms. Their experimental results indicated that the average values of the mean absolute percent errors were much lower than those of the comparison models.

Although there are multi-objective genetic algorithm related works in other fields, the combination of side-channel analysis and a multi-objective genetic algorithm is seldom reported. Zhang et al. [18] put forward a non-profiling power attack based on the phenomenon that the correlation coefficient was related to the number of correct key bytes in a parallel implementation: the larger the number of correct key bytes, the higher the coefficient. As a result, they applied a genetic algorithm to turn the key searching problem into a Pearson correlation coefficient problem. They proposed a novel leakage model based on the power consumption of multiple S-boxes and the number of traces needed to recover was decreased. Ding et al. [19] found that Zhang’s method (SGA-CPA) was faced with premature convergence challenges when there were plenty of large S-boxes, for example AES-128 cryptographic algorithm. Ding et al. studied the cause of premature convergence and put forward an intelligent multiple sieve method which is called multiple sieve-CPA (MS-CPA) to solve the problem. Their experimental results showed that the success rate of MS-CPA was much better than SGA-CPA. However, none of them took multi-objective optimization into account.

In our past work, we confirmed that multi-byte electromagnetic analysis had better performance than single byte electromagnetic analysis. Compared with just one correct key byte, the correlation coefficient is higher when there are two or more correct key bytes. Our experiments also showed that when correct key bytes were summed, the correlation coefficient was clearly distinguishable, but when correct key bytes and incorrect key bytes were summed, the correlation coefficient was usually in mass. It was difficult to distinguish right from wrong.

Multi-objective optimization is a good way to solve the problem of a single byte of the key. So in this work, we will apply it to multi-byte electromagnetic analysis. A genetic algorithm is well suited to solve multi-objective optimization problems because of its population-based approach, and it is also used in our method. Eventually, we put forward a novel multi-objective electromagnetic analysis method based on a genetic algorithm and obtained better performance.

### 2.2. Cryptographic Algorithm and Hamming Distance Model

The Advanced Encryption Standard (AES) [23,24] is a symmetric encryption algorithm which can process data using cipher keys with 128, 192 or 256 bits. In our experiment, AES-128 encryption algorithm is used for the analysis. AES-128 encryption algorithm consists of 10 rounds, acting on a 128-bit block represented as a state consisting of 16 bytes. Every round is made up of 4 operations except the last round which skips the MixColumns operation:

**SubBytes**: it is a non-linear byte substitution that operates independently on each byte of the state using a substitution table;

**ShiftRows**: the bytes in every row of the State are cyclically shifted over different numbers of bytes. The *n*-th row is cyclically shifted over *n*-1 bytes;

**MixColumns**: this operation treats each column as a 4-byte vector and multiplies it by a constant matrix;

**AddRoundKey**: this operation adds a round key to the state by bitwise XOR operation.

**Figure 1:**
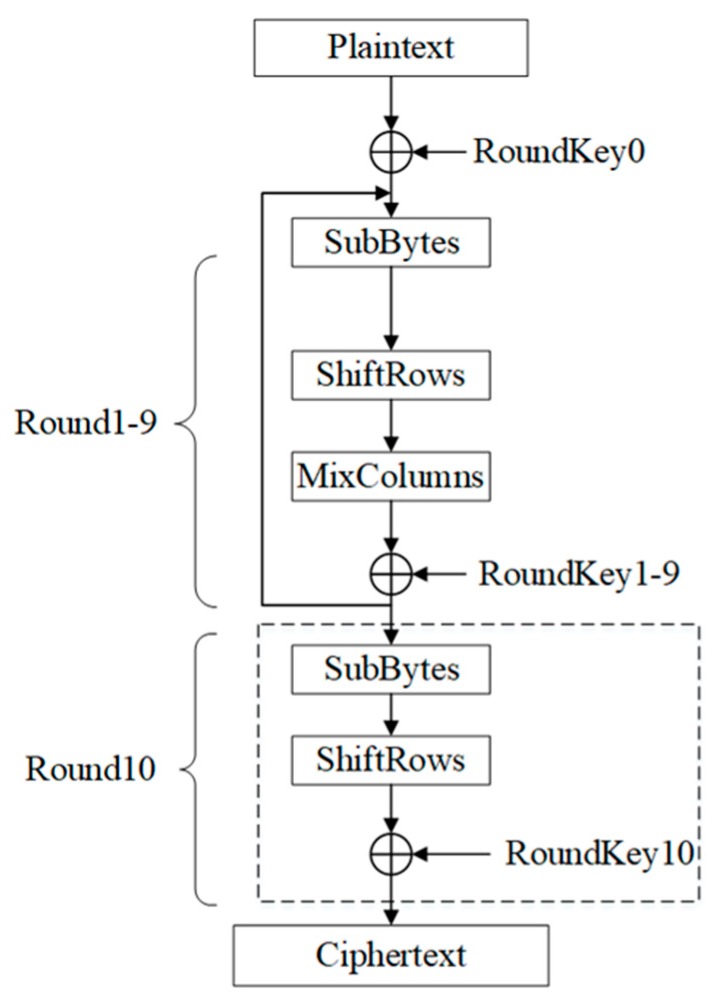
The main process of AES-128.

The main process of AES-128 is presented in Figure 1:

The plaintexts are masked with a 128 bits white key before the first round. At the end of every round, a 128-bit round key is XORed with a 128-bit intermediate value.

We know that when the encryption algorithm is implemented on cryptographic devices, there are some predictable relations between electromagnetic leakage and cryptographic operations. The hamming distance (HD) model was firstly proposed by Eric Brier et al. in 2004 [4] where they assumed that the leakage was dependent on the number of flipping bits. In a m-bit microprocessor, binary data is coded $X=\overset{m-1}{\underset{j=0}{\sum }}d_{j}2^{j}$, with the bit values $d_{j}$ = 0 or 1. So its hamming weight $HW\left(X\right)=\overset{m-1}{\underset{j=0}{\sum }}d_{j}$. If *X* contains *m* independent and uniformly distributed bits, the average hamming weight $\mu_{HW}=\frac{m}{2}$ and the variance $\sigma_{HW}^{2}=\frac{m}{4}$.

The hamming distance between two values *X* and *Y* can be calculated by the following equation:

(1) HD(X,Y)=HW(X⨁Y)

Hamming distance is the number of flipping bits to go from X to Y. It is similar to hamming weight which assumes that Y=0. So HW(X⨁Y) has the same mean $\frac{m}{2}$ and the variance $\frac{m}{4}$. The hamming distance model is more suitable to predict the relation, and we also applied it to our electromagnetic analysis.

From Figure 1, we can know that there are only three operations in the last round which is more feasible to recover the key by analyzing the electromagnetic leakage. The hamming distance model used in our method to estimate hypothetical electromagnetic leakage is:

(2) L=α·HD(X,Y)+β=α·HW(X⨁Y)+β

where *L* is electromagnetic leakage, HW(X⨁Y) is the hamming distance between *X* and *Y* which is known as the number of flipping bits between *X* and *Y*, *HW* is hamming weight, *α* is a scalar gain and *β* is usually considered as noise.

In order to depict the correlation in detail, the Pearson correlation coefficient is applied to predict the correlation between the hamming distance and the measured electromagnetic leakage.

(3) $$\rho_{L\;HW}=\;\frac{cov\left(L,HW\right)}{\sigma_{L}\sigma_{HW}}$$

where *cov*() is the covariance between *L* and *HW*, *σ* is the standard deviation.

The hamming distance model is a linear model, and we can predict the relationship between the variances of different variables:

(4) $$\sigma_{L}^{2}=\;\alpha^{2}\sigma_{HW}^{2}+\sigma_{\beta }^{2}$$

Under the uncorrelated noise assumption, the Pearson correlation coefficient is transformed to:

(5) $$\rho_{L\;HW}=\;\frac{\alpha \cdot \sigma_{HW}}{\sigma_{L}}=\;\frac{\alpha \cdot \sigma_{HW}}{\sqrt{\alpha^{2}\sigma_{HW}^{2}+\sigma_{\beta }^{2}}}=\;\frac{\alpha \sqrt{m}}{\sqrt{m\alpha^{2}+4\sigma_{\beta }^{2}}}$$

We can guess the unknown key bits and calculate *ρ* for every key candidate. The maximum |*ρ*| is considered as the correct key.

In a real scenario, a set of *N* electromagnetic traces *T_i_* and *N* associated random plaintexts, the estimate correlation factor $\rho_{T\;HW}$ is given by the following formula:

(6) $$\rho_{T\;HW}=\frac{N\sum^{\;}T_{i}HW_{i\;}-\sum^{\;}T_{i}\sum^{\;}HW_{i}}{\sqrt{N\sum^{\;}T_{i}^{2}-{\left(\sum^{\;}T_{i}\right)}^{2}}\sqrt{N\sum^{\;}HW_{i}^{2}-{\left(\sum^{\;}HW_{i}\right)}^{2}}}$$

where the summations are taken over the N samples (*i* = 1,···, *N*) at every time step within the electromagnetic traces *T_i_*(*t*).

In theory, it is difficult to compute the variance of $\rho_{T\;HW}$ with the number of available samples *N*. In practice, a few hundred experiments is enough to provide an approximate estimate of the correlation. Further detailed information can be referred to [25] which shows the method can be regarded as a maximum likelihood model fitting procedure.

### 2.3. Genetic Algorithm

Inspired by Darwin’s theory of natural selection and evolutionary biology, the genetic algorithm (GA) [26,27] is a heuristic search method applied in optimization problems. GA employs a repeated process of selection, crossover and mutation of potential solutions in search of the optimal one for the problem.

There are some basic elements we need to know in a simple genetic algorithm:

- **Individual**. The potential key candidates to the optimization problem are regarded as individuals.
- **Fitness**. The objective function to evaluate the fitness of an individual is regarded as the fitness function.
- **Population**. The population is a group of individuals initialized randomly.
- The simple genetic algorithm is mainly composed of three operations:
- **Selection**. This operator selects individuals in the population for reproduction. The fitter the individual, the more times it is likely to be selected.
- **Crossover**. This operator exchanges key bits between two individuals selected randomly with the probability *P_c_* to generate new individuals.
- **Mutation**. This operator randomly flips some bits in an individual with a lower probability *P_m_* to generate new individuals.

The mathematical basis of genetic algorithm is Holland’s schema theorem which describes that a particular schema *H* receives more copies in the offspring by the operation of selection, crossover, and mutation.

**Schema theorem**: Under the influence of selection, crossover and mutation operations, short, low-order schemata with above-average fitness increase exponentially in frequency in successive generations.

(7) $$m\left(H,t+1\right)=m\left(H,t\right)\frac{f\left(H\right)}{\bar{f}}$$

(8) $$m\left(H,t+1\right)\geq m\left(H,t\right)\frac{f\left(H\right)}{\bar{f}}\left[1-\frac{P_{c}\cdot \delta \left(H\right)}{m-1}\right]\left[\;1-o\left(H\right)P_{m}\right]$$

where *m*(*H*,t) represents that there are m examples of a particular schema *H* contained within the population at a given time step t; f(H) is the average fitness of the strings representing schema *H* at time t; $\bar{f}$ is the average fitness of the strings representing all individuals at time *t*,  o(H) is the order of the schema *H*,  δ(H) is the defining length of the schema *H*, $P_{c}$ is the crossover rate, and $P_{m}$ is the mutation rate.

We can see that if f(H) is greater than $\bar{f}$, then *m*(*H*,t+1) will be greater than *m*(*H*,t). In a nutshell, the schema with above-average fitness will receive an increasing number of samples in the offspring while the schema with below-average fitness will receive a decreasing number of samples in the offspring.

In [18], Zhang et al. put forward a method based on a phenomenon that the correlation coefficient was related to the number of correct bytes of the key. Hence, they transformed this to find the optimal correlation coefficients of key candidates instead of the problem of searching for the correct key. In a simple genetic algorithm, the key candidates were defined as individuals and the Pearson correlation coefficient is defined as fitness.

The main steps of a simple genetic algorithm in our experiment are shown in Algorithm 1:
**Algorithm 1** Simple Genetic Algorithm**Input:** max generation *gen_max*, size of population *NIND*, crossover rate *P_c_*, mutation rate *P_m_* **Output:** the optimal solution 1: *key_cand* = Initialization(*NIND*); 2: Fitness(*key_cand*); 3: *gen* = 0; 4: *key_right* = 0; 5: **while**
*gen < gen_max **and** key_right = = 0 **do*** 6:   Selection(*key_cand*); 7:   Crossover(*key_cand*,*P_c_*); 8:   Mutation(*key_cand*,*P_m_*); 9:   Fitness(*key_cand*); 10:  *key_optimal* = MaxFitness(*key_cand*); 11:  *gen* = *gen* + 1; 12:  **if** Vertification(*key_optimal*) = **true then** 13:   *key_right* = 1 14:  **end if** 15: **end while** 16: **return**
*key_optimal*

### 2.4. Multi-Objective Optimization

Multi-objective optimization [28,29,30] is widely used in our daily life and most engineering optimization problems. It is an optimization problem of vector functions. The comparison of vector function values is more complicated than the comparison of scalar value. The optimal solution in the single-objective optimization problem is often only a non-inferior solution in the multi-objective optimization problem. In the ideal case, multi-objective optimization requires that each component objective is optimal.

There are many ways to solve multi-objective optimization problems. The most important method is to convert a multi-objective optimization solution into an appropriate one. It usually can be divided into two parts: one is to reconstruct a new objective function to convert a multi-objective optimization problem into a single-objective optimization problem; another is to transform the multi-objective optimization problem into a series of single-objective problems.

(9) $$\{\begin{array}{c} V-\max\;F\left(X\right)={\left[f_{1}\left(x_{1}\right),f_{2}\left(x_{2}\right),\ldots ,f_{i}\left(x_{n}\right)\right]}^{T}\\ X=\left[x_{1},x_{2},\ldots ,x_{n}\right]\\ s.t.\;\;\;\;\;\;\;\;\;\;\;f_{i}\left(x_{n}\right)\in \left[-1,1\right]\\ \;x_{n}^{i}\in \left\{0,1\right\} \end{array}$$

where $f_{i}\left(x\right)$ is the Pearson correlation coefficient of the *i*-th byte of the key. We want to get the maximum F(X) which also means every $f_{i}\left(x\right)$ is supposed to be as large as possible.

In this paper, we adopted the second one as the solution to CEMA. The genetic algorithm is well suited to solve multi-objective optimization problems because of its population-based approach. Combining the advantages of the genetic algorithm and multi-objective optimization, we applied a multi-objective genetic algorithm to CEMA, which can be described as follows:

(1) All key candidates in the group are equally divided into sub-groups by the subkey objective function;
(2) Every subkey objective function is computed independently in the corresponding sub-group;
(3) Individuals with high fitness in every sub-group are selected to form a new group;
(4) Crossover and mutation are performed in the new group;
(5) The sub-groups are recombined and the optimal one is found for multi-objective optimization.

**Figure 2:**
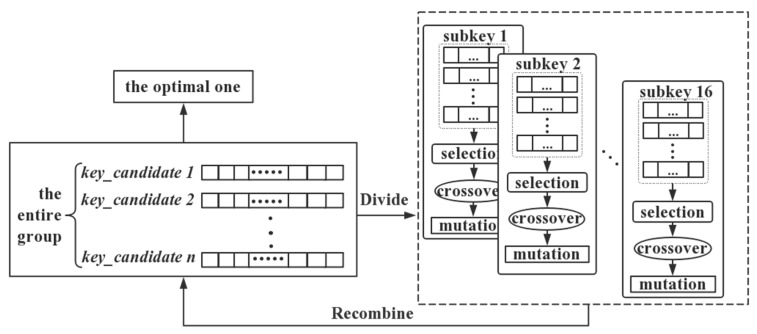
The basic schematic diagram of multi-objective optimization.

The main procedure of multi-objective genetic algorithm to CEMA is shown in Figure 2:

## 3. Multi-Objective Electromagnetic Analysis Based on Genetic Algorithm (MOGAEMA)

### 3.1. MOGAEMA

Our multi-objective electromagnetic analysis based on genetic algorithm (MOGAEMA) is used as an alternative to CEMA using multi-bytes as the objective instead of one byte to recover the key. The advantage of multi-objective electromagnetic analysis is that it takes full advantage of information contained in all bytes of the key. In this part, we illustrate the multi-objective electromagnetic analysis based on a genetic algorithm when recovering the key in detail.

In MOGAEMA, the multi-objective function F(X) is defined as the fitness, and the single objective function $f_{i}\left(x\right)$ is defined as sub-fitness. The individuals are selected by fitness which is calculated by all bytes of the key. Sort and sieve operators are added to find the optimal situation. The sort operator will sort the sub-fitness in descending order, better subkey candidates in different groups will be ranked in the front, so that better subkey candidates can be combined together with greater probability to generate optimal key candidates. For the sieve operator, we have modified sieve operator in [19] and applied it into our method to find the best key candidate when better key candidates in every generation, which we called key optimal candidates, are selected. We select a key optimal candidate as *key_temp* and every byte of the key optimal candidates will be replaced if they are different from *key_temp*. The fitness of *key_temp* will be recomputed, if the fitness is larger than the previous one, *key_temp* will be updated and maintained until the last one is compared. As a result, *key_temp* has better fitness than others.

The main procedure of MOGAEMA is shown in Algorithm 2:
**Algorithm 2** Multi-Objective Genetic Algorithm**Input:** max generation *gen_max*, size of population *NIND*, crossover rate *P_c_*, mutation rate *P_m_* **Output:** the optimal solution 1: *key_cand* = Initialization(*NIND*); 2: *subkey_cand* = Divide(*key_cand*) 3: SubFitness(*subkey_cand*); 4: *gen* = 0; 5: *key_right* = 0; 6: **while**
*gen* < *gen_max*
**and**
*key_right* = = 0 **do** 7:   Selection(*subkey_cand*); 8:   Crossover(*subkey_cand*,*P_c_*); 9:   Mutation(*subkey_cand*,*P_m_*); 10:  SubFitness(*subkey_cand*); 11:  Sort(*subkey_cand*) 12:  Recombine(*subkey_cand*); 13:  Fitness(*key_cand*); 14:  Selection(*key_cand*); 15:  *key_optimal_cand* = MaxFitness(*key_cand*) 16:  *gen* = *gen* + 1; 17:  **if** Vertification(*key_optimal_cand*) = **true then** 18:   *key_optimal* = *key_optimal_cand*; 19:   *key_right*=1; 20:  **endif** 21: **end while** 22: **if** (*key_right*=0) **then** 23:  *key_optimal* = Sieve(*key_optimal_cand*) 24: **end if** 25: **return**
*key_optimal*

### 3.2. MOGAEMA Experimental Platform

**Figure 3:**
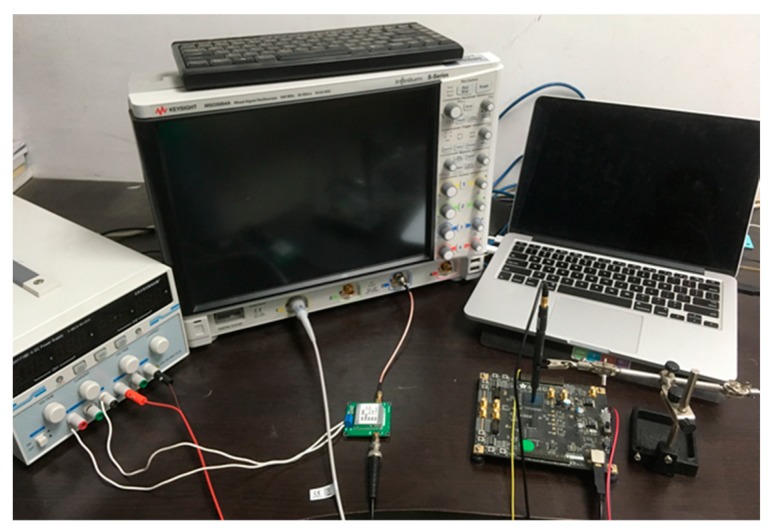
The multi-objective electromagnetic analysis based on genetic algorithm (MOGAEMA) experimental platform.

In this part, we introduce our experimental platform in detail. Our experiment with MOGAEMA was performed on a Sakura-G board [31], and the board is a universal test device to standardize the security evaluation methodology of cryptographic modules against side channel analysis. The MOGAEMA experimental platform is shown in Figure 3. We encrypted random plaintexts with a fixed key using AES-128 implemented on Sakura-G board. Original electromagnetic leakage traces were acquired by a magnetoelectric sensor first and then transmitted to the oscilloscope while AES was running on the Sakura-G board. A computer gave an automated setup for data collection and communication. The Sakura-G board is powered by a direct current (DC) power supply to reduce irrelevant influence.

## 4. Results

**Figure 4:**
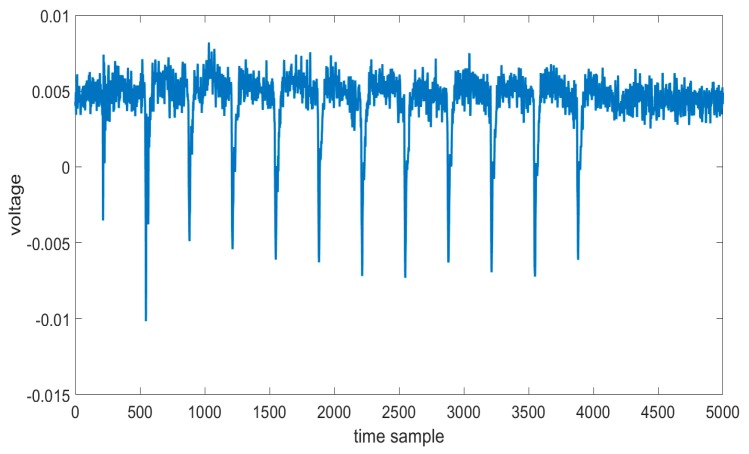
The original electromagnetic trace.

In this part, we show the efficiency of our method by different sets of traces. One electromagnetic leakage trace acquired by the sensor is shown in Figure 4, where we can clearly recognize every round of the AES-128 cryptographic algorithm. There are 12 peaks in the electromagnetic trace; the first peak which is different from the others, is caused by the trigger signal. The second peak is the loading of plaintext into the register of Sakura-G board and the following 10 peaks are 10 rounds of the AES-128 cryptographic algorithm.

**Figure 5:**
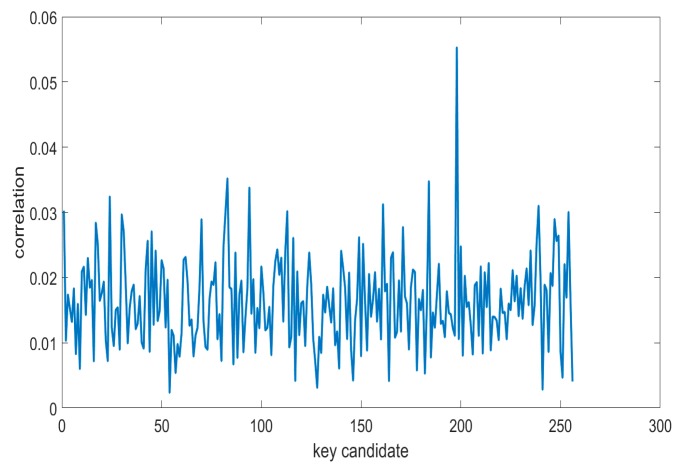
The correlation of subkey by correlation electromagnetic analysis (CEMA).

Correlation electromagnetic analysis (CEMA) is a more traditional and prevailing method in side-channel analysis. Its performance is much better than other methods in traditional side-channel analysis. In traditional CEMA, we only focus on one byte of the key to search for all possible subkey candidates. When the right subkey occurs, the correlation coefficient is largest if there are enough traces. We can obtain the correlation as shown in Figure 5. The picture shows that there is an obvious peak that is higher than the others in 197. The largest correlation coefficient is approximately 0.056 and most of others are lower than 0.03, so 197 is regarded as the right subkey.

**Figure 6:**
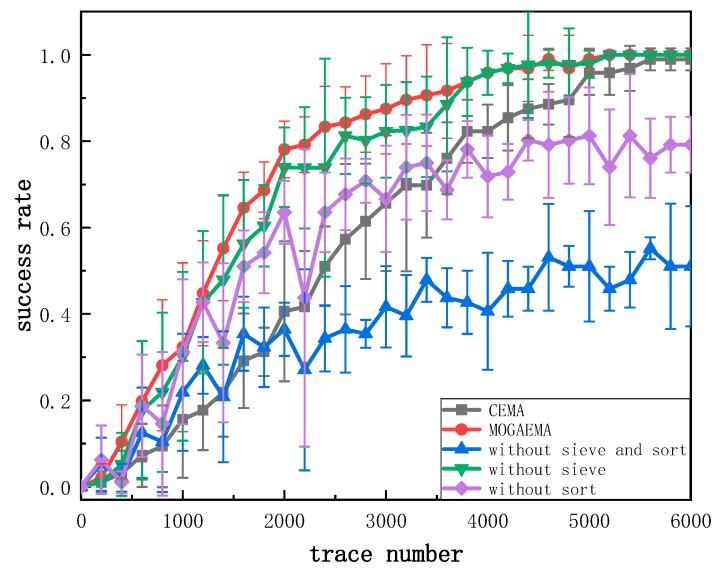
The performance of MOGAEMA with different operations.

The most popular method to evaluate performance is the success rate of the independent traces set. Consequently, we used success rate as a measure to test the performance of the MOGAEMA method. Operators are important factors affecting the performance of MOGAEMA. We have tested our MOGAEMA with different operators to verify which would be effective. Figure 6 shows the performance with five different operations: CEMA, MOGAEMA, MOGAEMA without sort operator, MOGAEMA without sieve operator, and MOGAEMA without sort and sieve operators.

The performance of MOGAEMA with sort and sieve operators is better than the others. Without the sieve operation, the performance is not stable during the process although it is better than CEMA. For the other two, their performance is worse than CEMA. We learn that the sieve operator makes the performance more stable in MOGAEMA and the sort operator plays an important role in recombining good subkey candidates to have good performance. Without the sort operator, subkey candidates are recombined randomly, and there is no relation between the key bytes. As a result, the fluctuation is larger than the others. So in the rest of the paper, we only consider MOGAEMA with sort and sieve operators.

**Figure 7:**
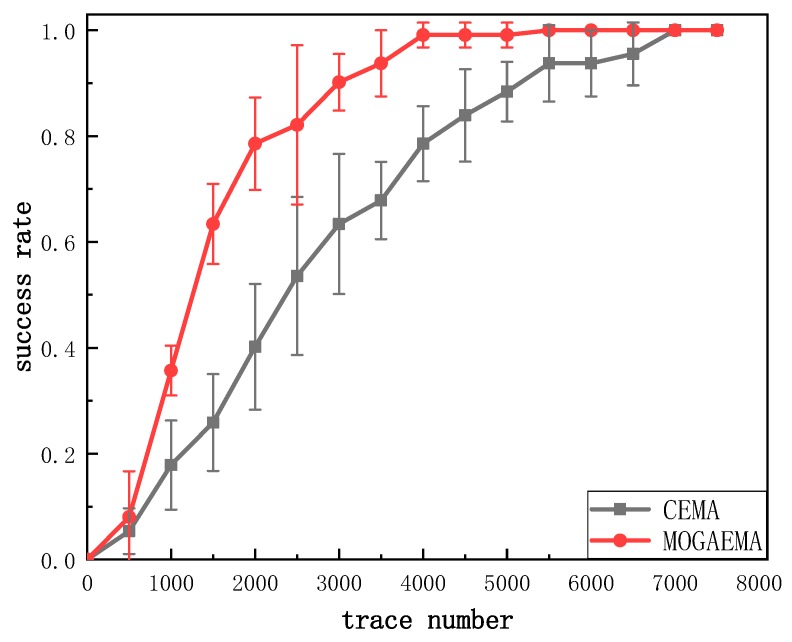
Success rate of CEMA and MOGAEMA.

In order to show the advantage of MOGAEMA over the existing CEMA method in terms of recovering the key, we compared the electromagnetic trace number required for MOGAEMA with traditional CEMA. Figure 7 shows the number of different methods when the key is recovered correctly. We can see that traditional CEMA requires about 7000 traces to reach success rate 1 while our method requires about 4000 traces.

**Figure 8:**
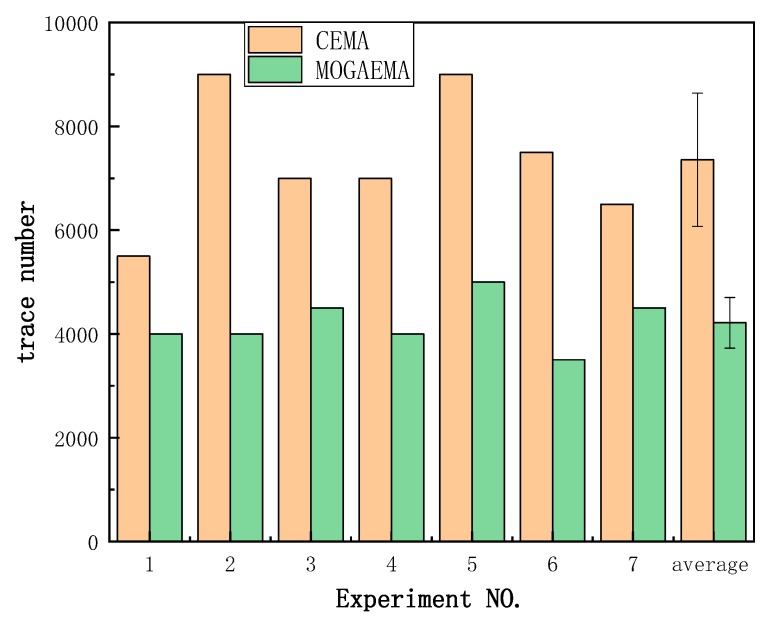
The minimum number of electromagnetic traces when the right key is recovered.

Besides this, we collected a large number of electromagnetic traces to verify our MOGAEMA method’s efficiency. We repeated the MOGAEMA method 7 times on different sets of traces and every set was selected at random. The minimum number of traces when we recovered the right key is summarized in Figure 8. The experimental result shows that the MOGAEMA method achieved greater improvement compared to CEMA in terms of success rate. According to a paired *t*-test, *p* value is $5.5\times 10^{-4}$. With the same number of traces, the MOGAEMA method obtained a higher success rate than CEMA. For example, the MOGAEMA method has recovered all bits of the key with 5000 traces while CEMA still need more traces to recover. On average, CEMA needs 7357 ± 1282 traces while MOGAEMA needs 4214 ± 488 traces. MOGAEMA achieved approximately 42.72% improvement for the corresponding case compared with CEMA.

We also applied our method to power trace analysis (which is called MOGAPA) to ensure efficiency. The power analysis experimental environment is similar to electromagnetic analysis. We also encrypted random plaintexts with a fixed key using the AES-128 cryptographic algorithm on the Sakura-G board.

**Figure 9:**
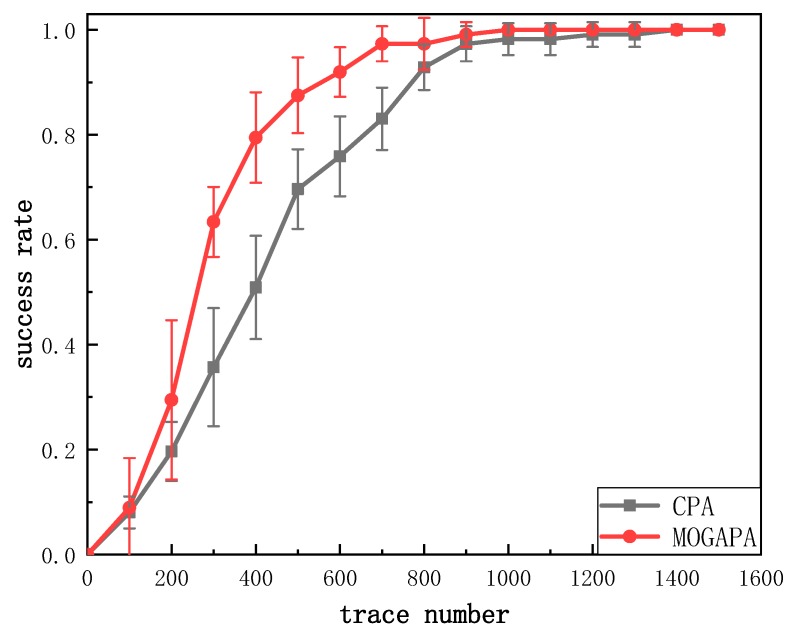
Success rate of correlation power analysis (CPA) and MOGAPA.

The success rate under different numbers of traces is shown in Figure 9. All bytes of the key were recovered with about 900 traces while traditional CPA still requires more than 300 traces.

**Figure 10:**
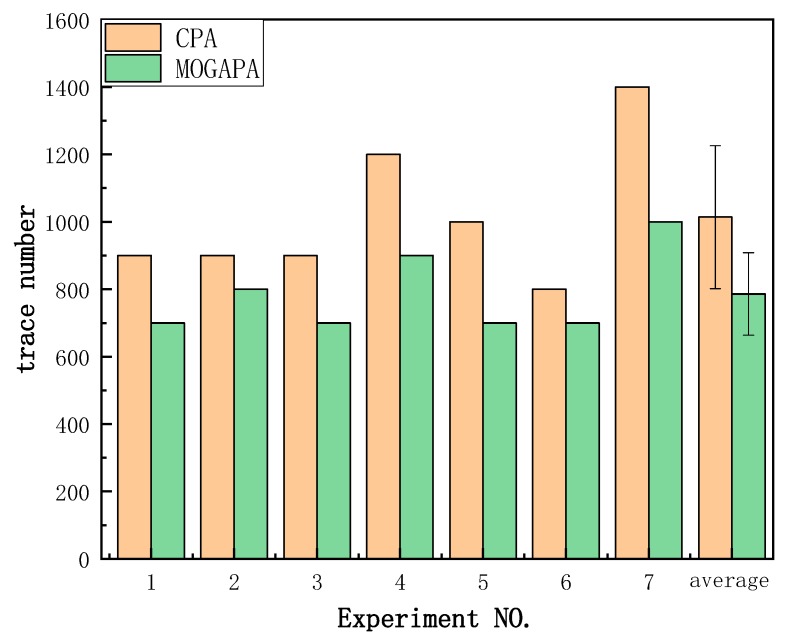
The minimum number of power traces when right key is recovered.

We also repeated our method on different sets of power traces selected at random. The minimum number of power traces when recovering the right key is summarized in Figure 10. We learn that our method is also suitable for power traces analysis. When recovering the key correctly, our method use fewer traces than traditional CPA. From Figure 10, we can see that our method reduced 100~400 traces in different experiments. According to the paired *t*-test, *p* value is $1.6\times 10^{-3}$. On average, CPA needs 1014 ± 212 traces while MOGAPA needs 786 ± 122 traces.

**Figure 11:**
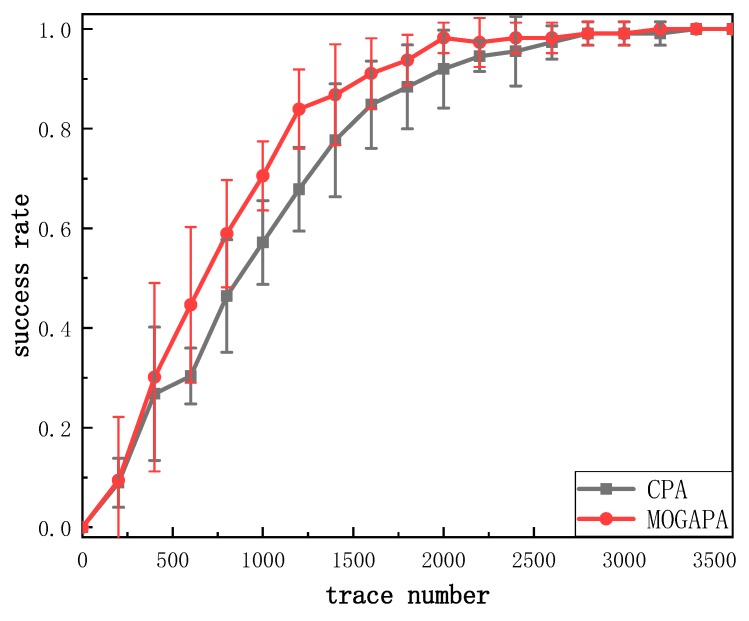
Success rate of CPA and MOGAPA.

**Figure 12:**
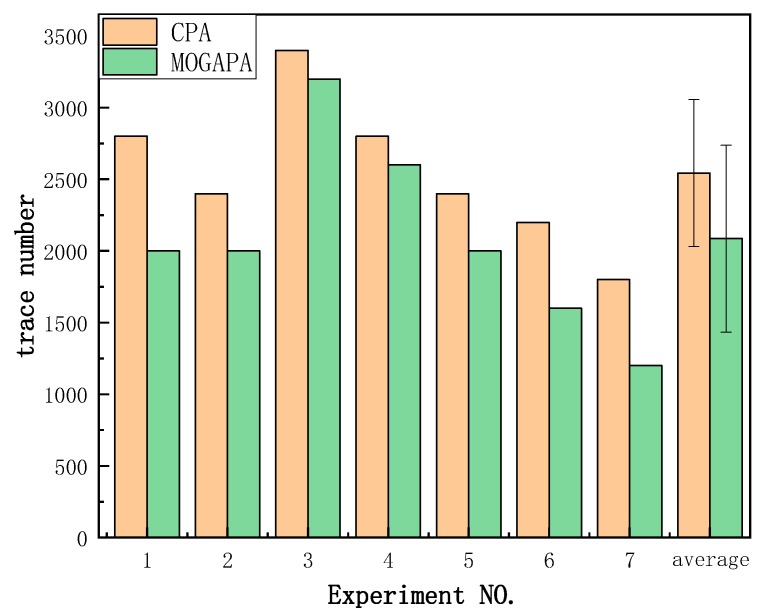
The minimum number of public traces when the right key is recovered.

In order to ensure the reproducibility of our experiments, we used a publicly available power traces dataset [32]. The NUEESS laboratory has implemented the unmasked AES on a Sasebo-GII board [33] provided by RCIS [34]. This board has a mechanism to provide users with different ways to access the reconfiguration function of FPGA. The performance of different experiments is shown in Figure 11 and Figure 12. We obtained the same conclusion as the previous experiments. Our method has a better performance than CPA which confirms the efficiency of our method. According to the paired *t*-test, *p* value is $1.6\times 10^{-3}$. On average, CPA needs 2543 ± 513 traces while MOGAPA needs 2086 ± 652 traces.

## 5. Conclusions and Future Work

CEMA is widely accepted to be an efficient method in side-channel analysis because of its simplicity and efficiency. However, CEMA only uses a single byte of the key which results in loss of information. In this paper, we put forward a novel multi-objective electromagnetic analysis method based on a genetic algorithm. In this way, we can take full advantage of information to obtain the key. As can be seen from the above sections, our method performed significantly better than the widely used CEMA in electromagnetic side-channel analysis. According to a paired *t*-test, *p* value is $5.5\times 10^{-4}$. In a MOGAEMA experimental environment, CEMA recovers the key with the trace number of 7357 ± 1282 on average while our method needs 4214 ± 488 traces. The trace number is reduced by approximately 42.72% for the corresponding case compared with CEMA on average.

However, there are also some imperfections while using multi-objective electromagnetic analysis based on a genetic algorithm. First, compared with power analysis, there is more noise in electromagnetic traces which causes a low signal-to-noise ratio, so we need more traces to recover the key. Second, there are many parameters in a genetic algorithm which are optimal parameters for different electromagnetic traces that still remain to be explored. Sometimes, premature convergence may cause failure in recovering the key which will be studied in future work.

In the future, research should aim to solve imperfections. First, we will try some new filtering works to improve the signal-to-noise ratio. Second, an adaptive parameters selection method of a genetic algorithm should be studied to find optimal parameters. In addition, cryptographic algorithms with countermeasures will be studied by our proposed method in the future.

## References

[1] Mangard S., Oswald E., Popp T. (2007). Power Analysis Attacks: Revealing the Secrets of Smart Cards.

[2] Kocher P.C. Timing Attacks on Implementations of Diffie-Hellman, RSA, DSS, and Other Systems. Proceedings of the Annual International Cryptology Conference.

[3] Kocher P.C., Jaffe J.M., Jun B.C. Differential Power Analysis. Proceedings of the 19th Annual International Cryptology Conference.

[4] Brier E., Clavier C., Olivier F. Correlation Power analysis with a leakage model. Proceedings of the Conference on Cryptographic Hardware and Embedded Systems 2004.

[5] Chari S., Rao J.R., Rohatgi P. Template Attacks. Proceedings of the Cryptographic Hardware and Embedded Systems 2002.

[6] Choudary M.O., Kuhn M.G. (2018). Efficient, Portable Template Attacks. IEEE Trans. Inf. Forensics Secur..

[7] Boneh D., Demillo R.A., Lipton R.J. On the importance of checking cryptographic protocols for faults. Proceedings of the International Conference on the Theory and Application of Cryptographic Techniques.

[8] Agrawal D., Archambeault B., Rao J.R., Rohatgi P. The EM Side-Channel(s). Proceedings of the Cryptographic Hardware and Embedded Systems 2002.

[9] Carlier V., Chabanne H., Dottax E., Pelletier H. Electromagnetic Side Channels of an FPGA Implementation of AES. https://eprint.iacr.org/2004/145.pdf.

[10] Gandolfi K., Mourtel C., Olivier F. Electromagnetic Analysis: Concrete Results. Proceedings of the Cryptographic Hardware and Embedded Systems 2001.

[11] Ding G., Chu J., Yuan L., Zhao Q. Correlation Electromagnetic Analysis for Cryptographic Device. Proceedings of the 2009 Pacific-Asia Conference on Circuits, Communications and Systems.

[12] Kasper T., Oswald D., Paar C. EM Side-Channel Attacks on Commercial Contactless Smartcards Using Low-Cost Equipment. Proceedings of the 10th Workshop on Information Security Applications.

[13] Li Y., Chen M., Wang J. Introduction to side-channel attacks and fault attacks. Proceedings of the Asia-Pacific International Symposium on Electromagnetic Compatibility (APEMC).

[14] Hospodar G., Gierlichs B., De Mulder E., Verbauwhede I., Vandewalle J. (2011). Machine learning in side-channel analysis: A first study. J. Cryptogr. Eng..

[15] Lerman L., Bontempi G., Markowitch O. (2014). Power analysis attack: An approach based on machine learning. IJACT.

[16] Sun S., Zhang H., Du Y. The electromagnetic leakage analysis based on arithmetic operation of FPGA. Proceedings of the 5th International Symposium on Electromagnetic Compatibility.

[17] Picek S., Samiotis I.P., Kim J., Heuser A., Bhasin S., Legay A.J.S. On the Performance of Convolutional Neural Networks for Side-channel Analysis. Proceedings of the International Conference on Security, Privacy, and Applied Cryptography Engineering.

[18] Zhang Z., Wu L., Wang A., Mu Z., Zhang X. (2015). A novel bit scalable leakage model based on genetic algorithm. Secur. Commun. Netw..

[19] Ding Y., Wang A., Yiu S.M. (2019). An Intelligent Multiple Sieve Method Based on Genetic Algorithm and Correlation Power Analysis. IACR Cryptol. Eprint Arch..

[20] Li J.-Q., Sang H.-Y., Han Y.-Y., Wang C.-G., Gao K.-Z. (2018). Efficient multi-objective optimization algorithm for hybrid flow shop scheduling problems with setup energy consumptions. J. Clean. Prod..

[21] Amoozegar M., Minaei-Bidgoli B. (2018). Optimizing multi-objective PSO based feature selection method using a feature elitism mechanism. Expert Syst. Appl..

[22] Du P., Wang J., Guo Z., Yang W. (2017). Research and application of a novel hybrid forecasting system based on multi-objective optimization for wind speed forecasting. Energy Convers. Manag..

[23] Joan Daemen V.R. (2002). The Design of Rijndael: AES—The Advanced Encryption Standard.

[24] Standard N.F. (2001). Announcing the advanced encryption standard (AES). Fed. Inf. Process. Stand. Publ..

[25] Brier E., Clavier C., Olivier F. (2003). Optimal Statistical Power Analysis. IACR Cryptol. Eprint Arch..

[26] Srinivas M., Patnaik L.M. (1994). Genetic algorithms: A survey. Computer.

[27] Goldberg D.E. (1989). Genetic Algorithms in Search, Optimization, and Machine Learning.

[28] Pettersson F., Chakraborti N., Saxen H. (2007). A genetic algorithms based multi-objective neural net applied to noisy blast furnace data. Appl. Soft Comput..

[29] Konak A., Coit D.W., Smith A.E. (2006). Multi-objective optimization using genetic algorithms: A tutorial. Reliab. Eng. Syst. Saf..

[30] Fonseca C.M., Fleming P.J. Genetic Algorithms for Multiobjective Optimization: Formulation Discussion and Generalization. Proceedings of the International Conference on Genetic Algorithms.

[31] SAKURA Hardware Security Project. http://satoh.cs.uec.ac.jp/SAKURA/hardware/SAKURA-G.html.

[32] TeSCASE Group. http://tescase.coe.neu.edu/?current_page=POWER_TRACE_LINK.

[33] Evaluation Environment for Side-Channel Attacks. https://www.risec.aist.go.jp/project/sasebo/.

[34] Side-Channel Attack Standard Evaluation Board (Sasebo): Sasebo-Gii. http://www.rcis.aist.go.jp/special/SASEBO/SASEBOGII-en.html.

